# Discovery of an enzyme and substrate selective inhibitor of ADAM10 using an exosite-binding glycosylated substrate

**DOI:** 10.1038/s41598-016-0013-4

**Published:** 2016-12-05

**Authors:** Franck Madoux, Daniela Dreymuller, Jean-Phillipe Pettiloud, Radleigh Santos, Christoph Becker-Pauly, Andreas Ludwig, Gregg B. Fields, Thomas Bannister, Timothy P. Spicer, Mare Cudic, Louis D. Scampavia, Dmitriy Minond

**Affiliations:** 1 Lead Identification Division, Translational Research Institute, The Scripps Research Institute, 130 Scripps Way, Jupiter, FL 39453 United States; 2 Institute of Pharmacology and Toxicology, RWTH Aachen University, Wendlingweg 2, 52074 Aachen, Germany; 3 Department of Chemistry and Biochemistry, Florida Atlantic University, 5353 Parkside Drive, Jupiter, FL 39453 United States; 4 Torrey Pines Institute for Molecular Studies, 11350 SW Village Parkway, Port Saint Lucie, FL 34987 United States; 5 University of Kiel, Institute of Biochemistry, Unit for Degradomics of the Protease Web, Rudolf-Höber-Str. 1, 24118 Kiel, Germany; 6 Department of Molecular Therapeutics, Translational Research Institute, The Scripps Research Institute, 130 Scripps Way, Jupiter, FL 39453 United States; 7 Department of Drug Discovery and Development, Harrison School of Pharmacy, 3211B Walker Building, Auburn University, Auburn, Alabama 36849 USA; 8 New York University, New York City, NY 10027 USA

## Abstract

ADAM10 and ADAM17 have been shown to contribute to the acquired drug resistance of HER2-positive breast cancer in response to trastuzumab. The majority of ADAM10 and ADAM17 inhibitor development has been focused on the discovery of compounds that bind the active site zinc, however, in recent years, there has been a shift from active site to secondary substrate binding site (exosite) inhibitor discovery in order to identify non-zinc-binding molecules. In the present work a glycosylated, exosite-binding substrate of ADAM10 and ADAM17 was utilized to screen 370,276 compounds from the MLPCN collection. As a result of this uHTS effort, a selective, time-dependent, non-zinc-binding inhibitor of ADAM10 with K_i_ = 883 nM was discovered. This compound exhibited low cell toxicity and was able to selectively inhibit shedding of known ADAM10 substrates in several cell-based models. We hypothesize that differential glycosylation of these cognate substrates is the source of selectivity of our novel inhibitor. The data indicate that this novel inhibitor can be used as an *in vitro* and, potentially, *in vivo*, probe of ADAM10 activity. Additionally, results of the present and prior studies strongly suggest that glycosylated substrate are applicable as screening agents for discovery of selective ADAM probes and therapeutics.

## Introduction

A disintegrin and metalloprotease (ADAM, adamalysins) enzymes are implicated in various diseases, most prominently in cancer^1,2^ and neurodegenerative conditions^3^. The two best studied adamalysins, ADAM10 and ADAM17, have been shown to contribute to the acquired drug resistance of HER2-positive breast cancer in response to trastuzumab^4–6^. ADAM17 was shown to play a key role in maintaining HER2 phosphorylation during trastuzumab therapy, while ADAM10 levels increased in response to trastuzumab in cells and *in vivo*. Higher ADAM10 levels correlated with decreased clinical response to trastuzumab. Inhibition of ADAM10 activity or expression resulted in an increase of trastuzumab efficacy, which suggested that ADAM10 and ADAM17 are tractable targets for HER2-positive trastuzumab-resistant breast cancer.

Early metzincin inhibitor discovery relied on utilization of short, linear peptidic substrates that only interacted with active sites of target proteases. This strategy yielded multiple potent, broad-spectrum inhibitors of metzincins, some of which went as far as phase II and III clinical trials (e.g., marimastat, ilomastat), but ultimately failed due to either toxicity, poor oral bioavailability, metabolic stability issues, or lack of efficacy^7–9^. The majority of ADAM10 and ADAM17 inhibitor development has been focused on a discovery of inhibitors that bind the active site zinc^10,11^. However, in recent years there has been a shift from active site to secondary substrate binding site (exosite) inhibitor discovery^12,13^ which resulted in the identification of selective non-zinc binding inhibitors of ADAM17^12,14^.

There are several selective inhibitors of ADAM10 that are available to the researchers, including INCB8765 (Incyte Corporation, ADAM10 IC_50_ = 97 nM, ADAM17 IC_50_ = 2045 nM^15^), GI 254023X (Glaxo, ADAM10 IC_50_ = 5.3 nM, ADAM17 IC_50_ = 541 nM^11^), and ADAM10 prodomain (Biozyme Inc., ADAM10 IC_50_ = 48 nM, ADAM17 IC_50_ > 10 µM^16^). INCB8765 and GI254023X are small molecules containing hydroxamate moieties and, therefore, likely to inhibit ADAM10 *via* a zinc-binding mechanism^17^. ADAM10 prodomain is a competitive inhibitor of ADAM10, but it is unknown whether it binds the active site zinc. While zinc-binding inhibitors can exhibit a degree of selectivity between closely related enzyme isoforms, they ultimately cannot selectively inhibit shedding of substrates.

Our research is focused on the discovery and characterization of non-zinc-binding inhibitors of metzincins utilizing exosite-binding peptide substrates. Previously, we reported the discovery of MMP-13 and ADAM17 selective, non-zinc-binding inhibitors as a result of using exosite-binding substrates in small and medium scale screening efforts^13,18,19^. In the present work we examined whether exosite-binding substrates could be used in ultra-high throughput screening (uHTS) to discover selective, non-zinc-binding inhibitors of ADAM10 and ADAM17.

## Results

### Assay Miniaturization and Primary HTS campaign

**Figure 1:**
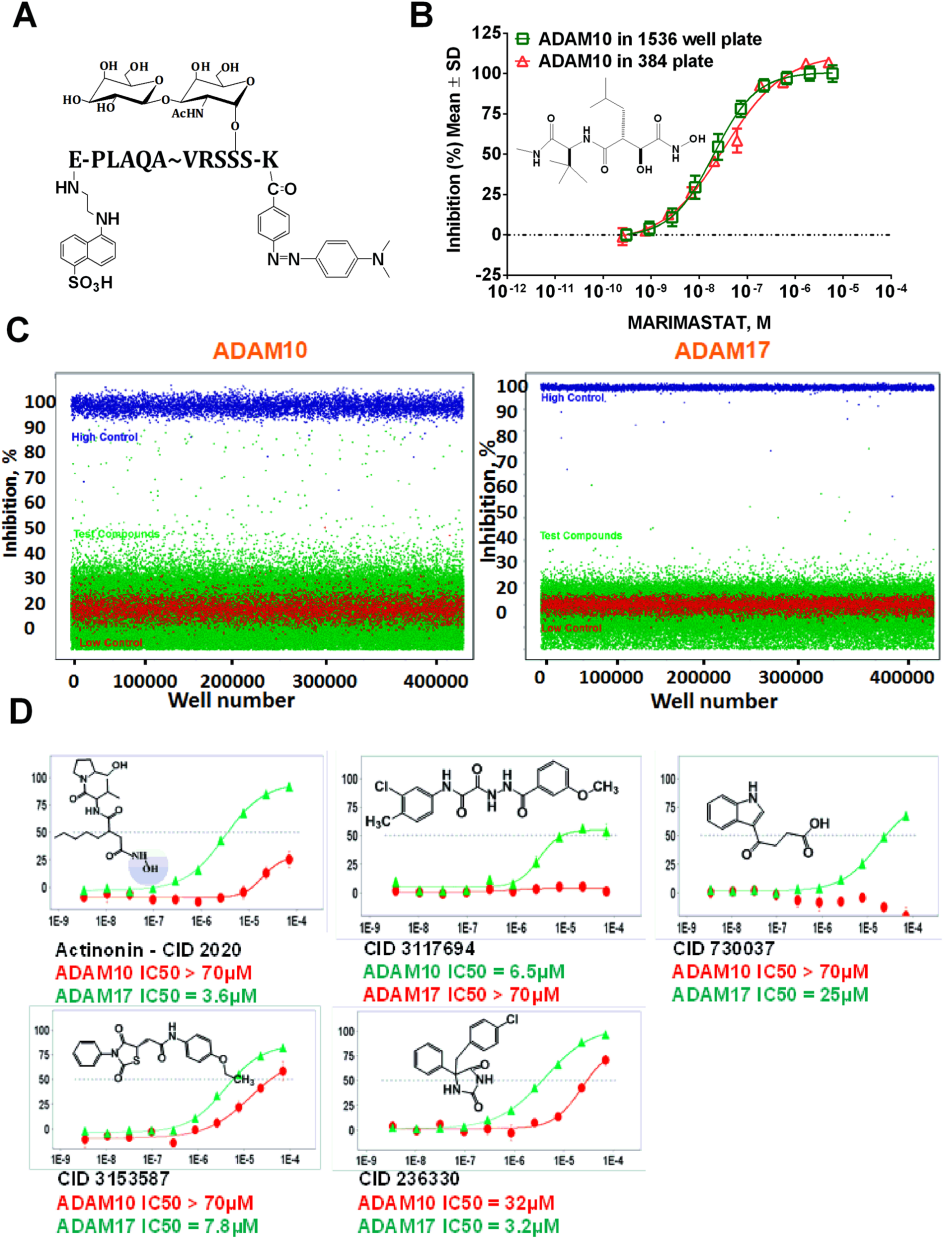
ADAM10 HTS assay. (**A**) Structure of glycosylated ADAM10 and ADAM17 uHTS substrate. (**B**) Pharmacological control (marimastat, structure shown on the graph) study with ADAM10 in 384 and 1536 well plate formats. (**C**) Scatter plot of ADAM10 and ADAM17 primary uHTS campaign. Green dots–test compounds, blue dots–100% inhibition control (marimastat at 10 µM), red dots–0% inhibition control (wells with uninhibited enzymatic reaction). (**D**) Examples of structures and dose response curves of HTS hits selected for follow-up studies. Hydroxamate is shown in a blue circle. Y-axis-%inhibition, X-axis–molar concentration of inhibitor.

To enable uHTS both ADAM10 and ADAM17 glycosylated substrate^13^ (Fig. 1A) assays were miniaturized to 1536 well plate format. The workflow of the assays in 384 well plate format was recaptured in 1536 well plate format and assay volume was scaled down to 5 µL. As an example, ADAM10 assay HTS in the 1,536 well plate format exhibited acceptable Z′, S/B, and %CV parameters similar to the ones in 384 well plate format. The IC_50_ value of marimastat (pharmacological control) was also reproducible between 384 and 1536 well plate formats (33 nM and 20 nM in 384 and 1536 well plate formats, respectively) (Fig. 1B). Primary uHTS campaigns for ADAM10 and ADAM17 were performed on 370,276 compounds from the Molecular Libraries Probe Center Network (MLPCN) collection^20^.

Both assays exhibited satisfactory QC parameters during the primary campaigns. The average Z′ values were 0.77 ± 0.04 and 0.90 ± 0.04 (n = 600 plates) for ADAM10 and ADAM17 campaigns, respectively. Average S/B values were 1.59 ± 0.06 and 3.16 ± 0.06 (n = 600 plates) for ADAM10 and ADAM17 campaigns, respectively.

Due to the high number of compounds giving negative % inhibition (Fig. 1C), an interval-based hit cutoff has been applied. This cutoff does not take into consideration compounds showing % inhibition higher than the average + 3SD of the high controls or % inhibition lower than the average–3SD of the low controls. Using interval-based hit cutoff of 19.78% and 8.62%, 2,294 and 3,080 hits were found for ADAM10 and ADAM17 campaigns, respectively. This constituted 0.62% and 0.83% hit rates for ADAM10 and ADAM17 campaigns, respectively.

### Hit validation and prioritization

In order to confirm activity and selectivity of hits from the primary uHTS campaigns, both ADAM10 and ADAM17 uHTS assays were performed in triplicate using just the hit compounds. 2,125 out of 2,294 ADAM10 hits and 2,872 out of 3,080 primary ADAM17 hits were available. ADAM10 and ADAM17 hits from primary uHTS campaigns were tested in triplicate. Based on the confirmation and counter screens the 250 top compounds for each target (total of 500 compounds) that were active for either target and inactive against the counter-target were considered for dose response studies.

235 out of 250 compounds for ADAM10 and 248 out of 250 compounds for ADAM17 were commercially available. Compounds were tested as 10-point, 1:3 serial dilutions starting at 70 µM in parallel in triplicate in both the ADAM10 and ADAM17 assays. As a result of the dose response studies, 49 compounds exhibited IC_50_ values < 10 µM for ADAM10 and 8 compounds exhibited IC_50_ values <10 µM for ADAM17.

We prioritized confirmed primary HTS hits for follow-up studies using primary selectivity criteria of 10-fold difference in IC_50_ values between ADAM10 and ADAM17. Overall, 5 compounds satisfied this criterion, 2 for ADAM10 and 3 for ADAM17 (Fig. 1D). Out of 3 compounds selective for ADAM17, one was a known zinc-binding metzincin inhibitor (actinonin) (Fig. 1D). It was discarded from follow-up studies due to its broad-spectrum activity against multiple metzincins^21^.

**Figure 2:**
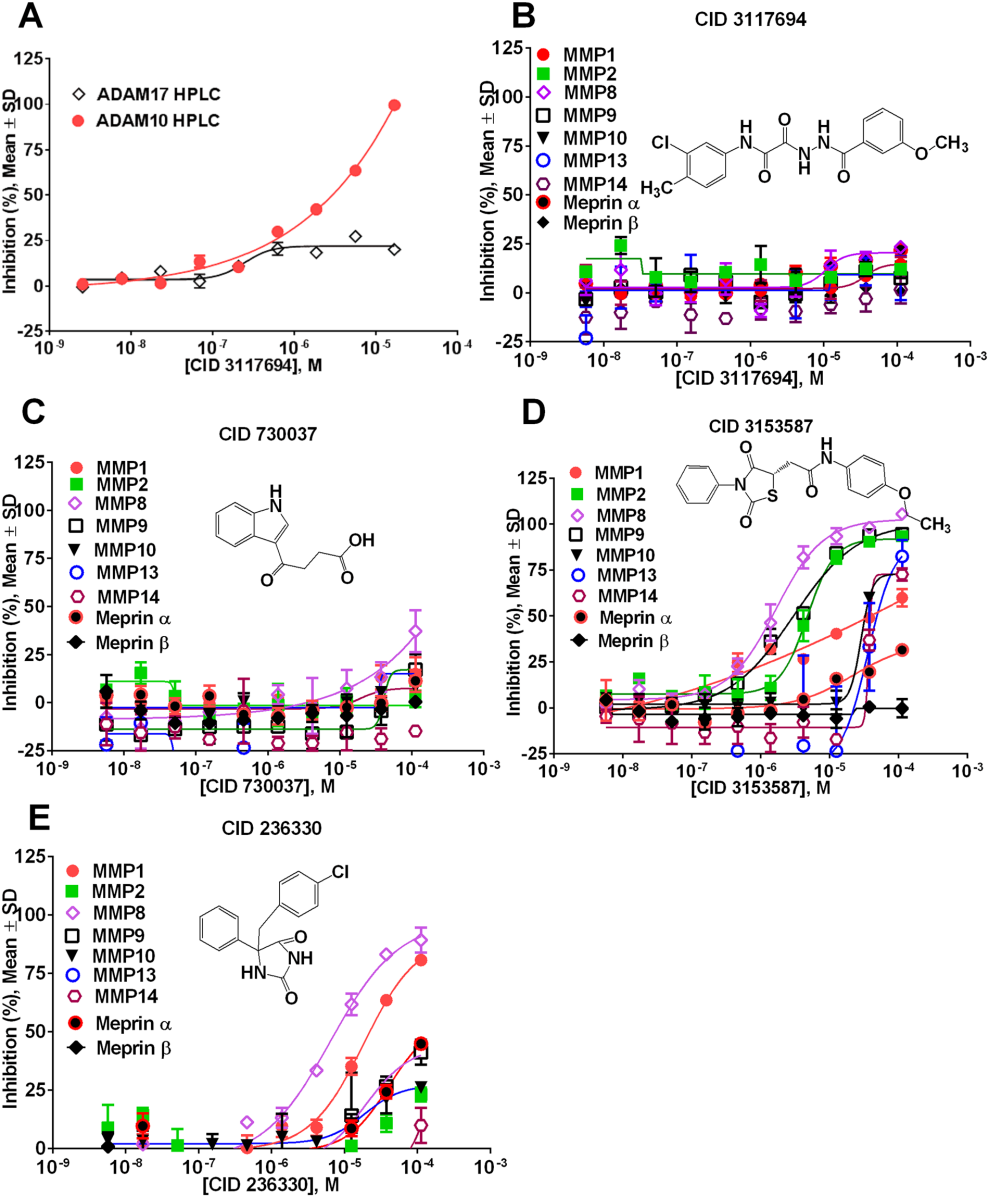
Results of dose response study of primary HTS hits against the panel of metzincins. (**A**) HPLC-based assays of CID 3117694 against ADAM10 and 17 using glycosylated substrate. (**B**) Screening of CID 3117694 against a panel of metzincins. (**C**) Screening of CID 730037 against a panel of metzincins. (**D**) Screening of CID 3153587 against a panel of metzincins. (**E**) Screening of CID 236330 against a panel of metzincins.

Powders for 4 compounds (CID # 3117694, 730037, 3153587, and 236330) were reordered from corresponding vendors and tested in 10-point, 1:3 serial dilutions starting at 100 µM in triplicate against ADAM10 and ADAM17 and a panel of metzincins. We examined the activity and selectivity of lead CID 3117694 using an ADAM10 and 17 HPLC-based assays using glycosylated substrate (Fig. 2A). IC_50_ values for ADAM10 inhibition were 4.1 µM and 1.1 µM in the HPLC and HTS assays, respectively, which is in agreement with the uHTS assay. Additionally, ADAM17-mediated hydrolysis of glycosylated substrate was not appreciably inhibited confirming the results of uHTS assay.

The reordered compounds confirmed their activity and selectivity for ADAM10 and ADAM17, with the exception of CID 730037 (Fig. 2C), which exhibited significantly lower activity against ADAM17 as compared to the original testing. Ultimately, CID 730037 was discarded from further testing. CID 3117694 exhibited somewhat greater potency than in the original screen (IC_50_ = 1.1 µM versus 6.5 µM). Testing of powders against a panel of metzincins revealed the selective nature of ADAM10 inhibition by CID 3117694 (Fig. 2B). Out of 11 tested metzincins (including ADAM10 and ADAM17), only ADAM10 was appreciably inhibited, which suggested that CID 3117694 could be inhibiting ADAM10 *via* a non-zinc-binding mechanism.

These data suggest that CID 3117694 is indeed a selective inhibitor of ADAM10.

### Mechanistic characterization of CID 3117694

**Figure 3:**
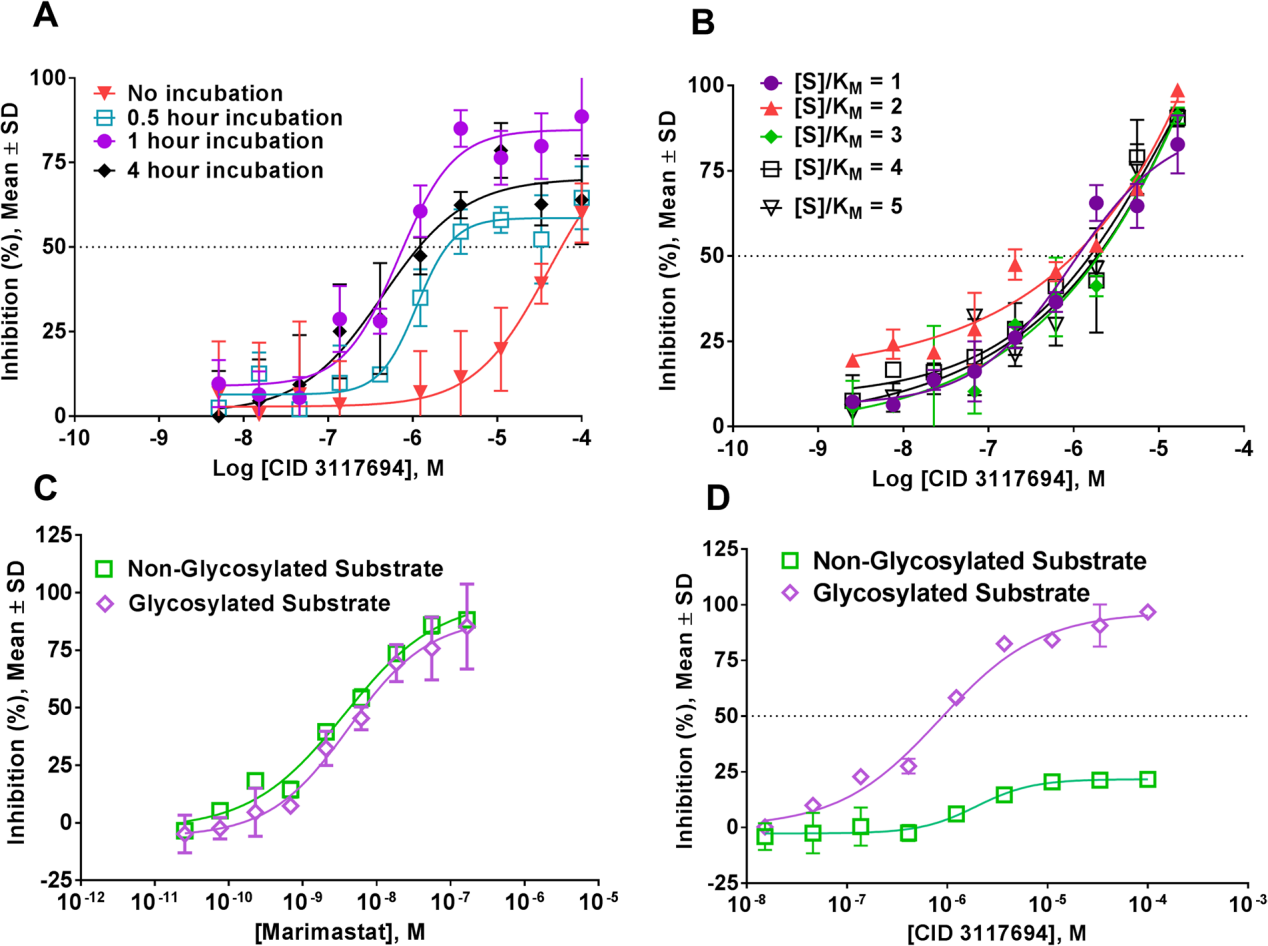
Effects of glycosylated and non-glycosylated substrates, [S], and time dependence on CID 3117694 inhibition of ADAM10 in endpoint assays. (**A**) Effect of CID 3117694 and ADAM10 pre-incubation time on CID 3117694 potency. (**B**) Effect of glycosylated substrate concentration on CID 3117694 potency. (**C**) Results of dose response study of marimastat with ADAM10 using glycosylated and non-glycosylated substrates. (**D**) Results of dose response study of CID 31176494 with ADAM10 using glycosylated and non-glycosylated substrates. Please note that active site zinc-binder marimastat inhibits both substrates equipotently, while CID 3117694 inhibits glycosylated substrate preferentially suggesting that CID 3117694 binds to an exosite of ADAM10 and not a Zn of an active site. ADAM10 was pre-incubated for 1 hour with CID 3117694.

We further characterized CID 3117694 in a series of assays to ascertain the type and modality of inhibition and the cell-based activity. We characterized the potency of CID 3117694 for dependence on enzyme and substrate concentration and incubation time with ADAM10. Potency and range of inhibition of CID 3117694 exhibited dependence on the pre-incubation time with ADAM10 (Fig. 3A). When ADAM10 was not pre-incubated with CID 3117694, the IC_50_ value was significantly greater than when the assay was run at uHTS condition (0.5 h pre-incubation), and it exhibited a partial inhibition profile (100% inhibition was not reached). Thus, IC_50_ values were 1.1 ± 0.1 µM and 40 ± 5 µM for 0.5 hour pre-incubation and no incubation, respectively, which constitutes an approximately 40-fold difference in the potency of CID 3117694. Longer incubation times (1 and 4 h) resulted in a slight increase of CID 3117694 potency as compared to uHTS conditions (IC_50_ = 1.1 ± 0.1, 0.7 ± 0.5, and 0.4 ± 0.3 µM, for 0.5, 1, and 4 h, respectively) suggesting that a potency plateau is reached at 1 h pre-incubation time. These data suggested that CID 3117694 is a time-dependent inhibitor of ADAM10, which, to our knowledge, has not been previously reported.

In addition to the time dependence, we characterized CID 3117694 for dependence on substrate concentration. CID 3117694 inhibited ADAM10 with similar IC_50_ values at all substrate concentrations (Fig. 3B). Lack of inhibition dependence on substrate concentration suggested that CID 3117694 is a non-competitive inhibitor of ADAM10. However, it is well-known that in the end-point assays, such as uHTS assays, time-dependent inhibitors can display a non-competitive behavior regardless of their true inhibition modality^22^.

CID 3117694 was tested for inhibition of ADAM10 in a non-glycosylated substrate assay. The preferential inhibition observed using a glycosylated, exosite-binding substrate compared with a non-glycosylated, active site-only binding substrate is a strong indication of an exosite inhibitor^18^. Marimastat, a known zinc binder, inhibited hydrolysis of the glycosylated substrate with the same potency as the hydrolysis of the non-glycosylated substrate (Fig. 3C). CID 3117694 inhibited hydrolysis of the glycosylated substrate significantly more potently than the non-glycosylated one (Fig. 3D; IC_50_ = 1.1 µM versus >100 µM, for glycosylated and non-glycosylated substrates, respectively), which suggested that CID 3117694 could be a non-zinc-binding inhibitor of ADAM10. There are no known non-zinc-binding inhibitors of ADAM10; therefore, these data suggest that CID 3117694 could be a first-in-class non-zinc-binding inhibitor of ADAM10. These data are in agreement with our previously published observation that a non-zinc-binding inhibitor of ADAM17, compound **15**, also preferentially inhibited hydrolysis of a glycosylated, exosite-binding substrate versus a non-glycosylated, active site-only binding substrate^23^. Interestingly, compound **15** exhibited unusual selectivity for a subset of ADAM17 substrates in cell-based assays^14^. This is an important feature of non-zinc-binding inhibitors, especially since ADAM10 sheds multiple cell-surface proteins and an indiscriminate inhibition of shedding could be detrimental for patients.

Since true affinity of time-dependent inhibitors cannot be determined by steady state approaches^24^, in order to determine the true inhibition modality and true affinity of CID 3117694 we performed kinetic assays with the range of glycosylated substrate (0.5–5 [S]/K_M_) and inhibitor (1.56–50 µM) concentrations in quadruplicate. Reactions were allowed to run for 6 h. Progress curve data were fitted using a non-linear regression model for time-dependent inhibition (Equation 1) using MatLab software.

**Figure 4:**
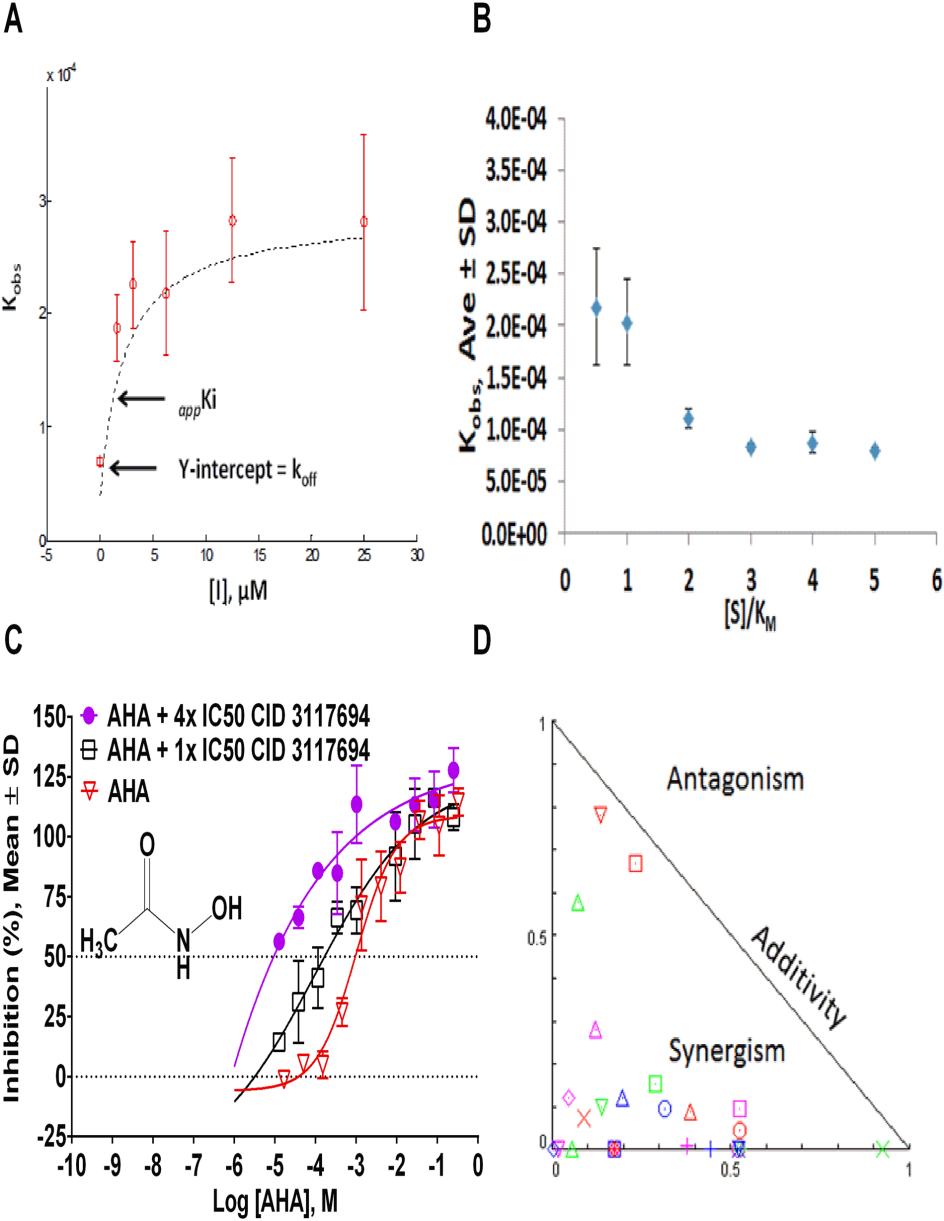
Mechanism of CID 3117694 inhibition of ADAM10. (**A**) Plot of K_obs_ versus [I] for CID 3117694 inhibition of ADAM10. Please note the hyperbolic relationship between k_obs_ and [I] suggesting that inhibitor binding complies with the two-step model. (**B**) Plot of K_obs_ versus [S]/K_M_ for CID 3117694 inhibition of ADAM10. Please note k_obs_ decreases with increase of [S]/K_M_ suggesting that inhibitor competes with a substrate. (**C**) Dose response study of ADAM10 inhibition by AHA alone and in the presence of varying dose of CID 3117694. Please note that AHA IC_50_ decreases significantly in the presence of CID 3117694. (**D**) Normalized isobologram of two drug combination effect on ADAM10 activity. Please note that majority of AHA-CID 3117694 combinations produced synergistic effect as compared to a single drug application.

First, we performed the ADAM10 reaction with glycosylated substrate in the presence of 15 µM CID 3117694 in kinetic format without pre-incubation. Obtained *k*_*obs*_ values were plotted versus [I] to determine whether the inhibitor binding conforms to a 1- or 2-step mechanism (Fig. 4A). The resulting curve of best fit was clearly hyperbolic (R^2^ = 0.997) suggesting the two-step binding model; therefore, we fitted data to Equation 2 to obtain the apparent potency of CID 3117694. Using a hyperbolic model, we derived _app_K_i_ and _app_K_i_* values of 1.36 ± 0.2 µM and 0.33 ± 0.02 µM, respectively, which approximated the IC_50_ value obtained in the endpoint uHTS assay experiment (IC_50_ = 1.1 ± 0.1 µM). Solving for K_i_ and K_i_′ using Equation 3 yielded the true potency (K_i_ = 0.883 µM and K_i_′ = 19 µM). The ratio of K_i_′/K_i_, designated as α (alpha), was 21.7, suggesting that CID 3117694 is a competitive inhibitor of ADAM10. In order to confirm the true binding modality, we plotted *k*_*obs*_ versus [S]/K_M_. *k*_*obs*_ decreased with increasing substrate concentration relative to K_M_ (Fig. 4B), corroborating that CID 3117694 is indeed a competitive inhibitor of ADAM10. Considering the lack of obvious zinc-binding moieties in the CID 3117694 structure, the competitive mode of ADAM10 inhibition suggests that binding occurs in one of the secondary substrate binding sites (exosites) that accommodates the glycosylated substrate. Given more potent inhibition of hydrolysis of the glycosylated substrate as compared to the non-glycosylated substrate (Fig. 3B), it also suggests that this is the exosite where only the glycosylated residue binds. Future studies will address the exact location of this exosite.

### Dual inhibition studies

In order to ascertain that CID 3117694 does not inhibit ADAM10 by binding to the active site zinc, we utilized a well-known zinc-binding inhibitor of metzincins, acetohydroxamic acid (AHA). AHA has been used in several dual inhibition studies^19,25^ as a zinc-binding probe due to its small size that virtually guarantees that it binds only to the zinc of an active site and does not interact with surrounding amino acid residues. AHA IC_50_ value for the inhibition of ADAM10-mediated hydrolysis of the glycosylated substrate was 1.0 ± 0.05 mM (Fig. 4C), reflecting the lack of interactions with amino acid residues. In the presence of 1.1 µM CID 31176944 (1.1 µM CID 31176944 = 1X IC_50_), the AHA IC_50_ value was 98 ± 5.2 µM, a 10-fold improvement in potency. Additionally, in the presence of 4.4 µM CID 31176944 (4.4 µM CID 31176944 = 4X IC_50_), the AHA IC_50_ value was 10 ± 1.5 µM, a 100-fold improvement in potency as compared to AHA alone. These data suggested that CID 3117694 and AHA act synergistically to inhibit ADAM10. Indeed, calculations of Combination Index (CI) (Tables 1 and 2) and isobolographic analysis (Fig. 4D) demonstrated that the majority of AHA/CID 3117694 combinations exhibit various degrees of synergism. Synergism between CID 3117694 and AHA suggests that compounds do not compete for the same binding site. Since AHA binds exclusively to the active site zinc this means that CID 3117694 does not. To our knowledge, CID 3117694 is a first non-zinc-binding inhibitor of ADAM10.

**Table 1:** Synergy assignment criteria based on Combination Index ranges.

CI	Description
<0.1	Very Strong Synergism (VSS)
0.1–0.3	Strong Synergism (SS)
0.3–0.7	Synergism (S)
0.7–0.85	Moderate Synergism (MS)
0.85–0.90	Slight Synergism (SlS)
0.90–1.10	Nearly Additive (NAdd)
1.10–1.20	Slight Antagonism (SlA)
1.20–1.45	Moderate Antagonism (MA)
1.45–3.3	Antagonism (A)
3.3–10	Strong Antagonism (SA)
>10	Very Strong Antagonism (VSA)

**Table 2:** Combination Index Summary for CID 3117694 and AHA.

CID, µM	AHA, µM	Effect	CI	Effect
4.4	83	0.999	0.52301	S
4.4	27.7	0.999	0.17455	SS
4.4	9	0.999	0.05672	VSS
4.4	3	0.999	0.01891	VSS
4.4	1	0.999	0.00631	VSS
2.2	83	0.999	0.52301	S
2.2	27.7	0.999	0.17455	SS
2.2	9	0.775	0.47897	S
2.2	3	0.764	0.23906	S
2.2	1	0.745	0.16928	SS
1.1	83	0.999	0.52300	S
1.1	27.7	0.999	0.17644	SS
1.1	9	0.919	0.47607	S
1.1	3	0.694	0.62195	S
1.1	1	0.663	0.32134	S
0.55	83	0.999	0.52489	S
0.55	27.7	0.999	0.17518	SS
0.55	9	0.984	0.44296	S
0.55	3	0.702	0.57227	S
0.55	1	0.542	0.44215	S
0.275	83	0.999	0.52300	S
0.275	27.7	0.999	0.17518	SS
0.275	9	0.957	0.92758	NAdd
0.275	3	0.788	0.38869	S
0.275	1	0.512	0.41083	S

CI–Combination Index, S–synergism, SS-strong synergism, VSS–very strong synergism, NAdd–nearly additive. See Table in the Materials and Methods for the determination of synergy ranges and CI calculations.

### Substrate selectivity profile of CID 3117694: HER2, CXCL16 and syndecan-4 shedding inhibition

**Figure 5:**
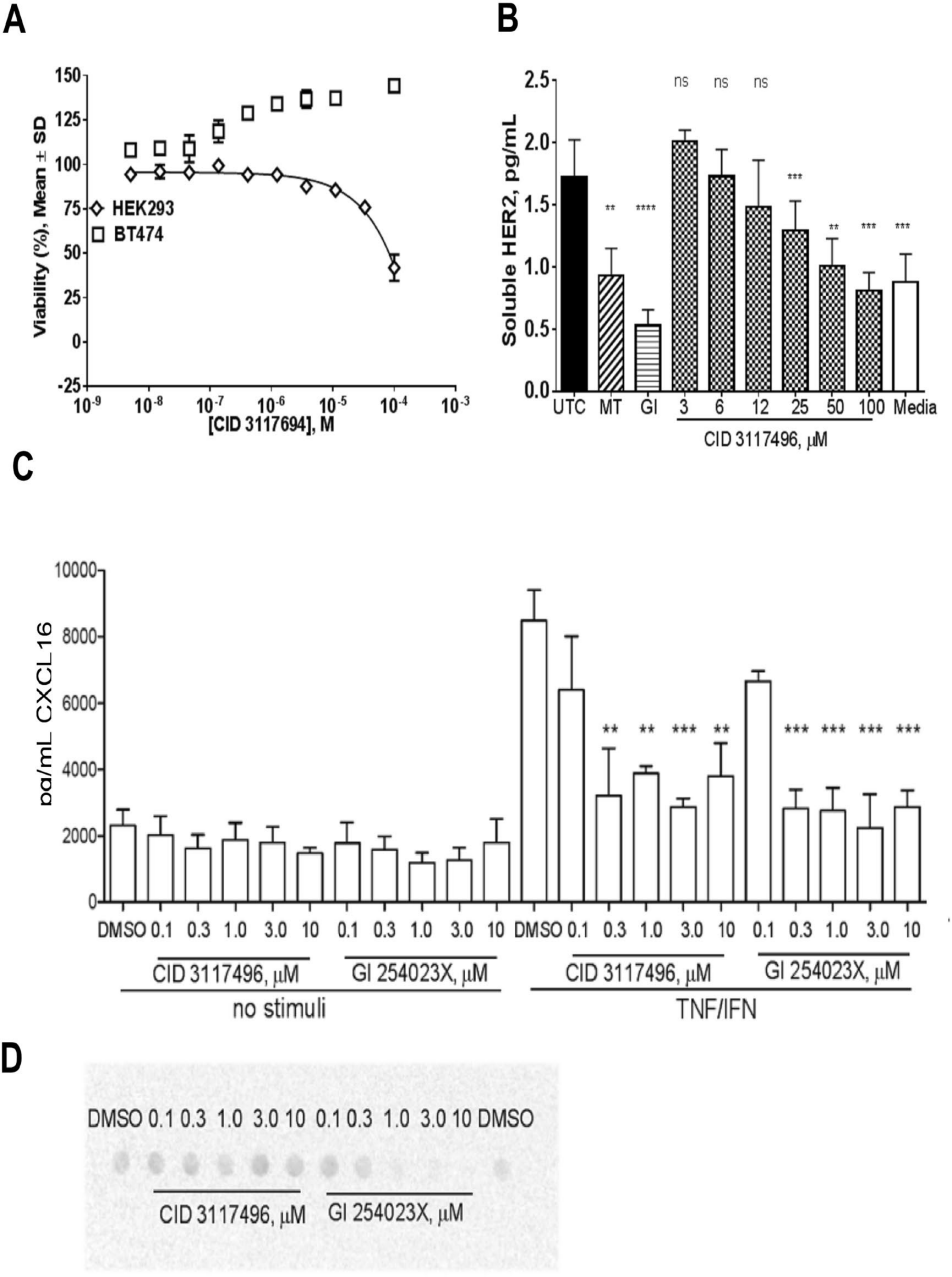
Inhibition of shedding of HER2, CXCL16 and syndecan by CID 3117694. (**A**) Effect of CID 3117694 on viability of BT474 breast cancer cells and HEK293 cells. Please note that only 100 µM of CID 3117694 has moderate effect on HEK293 cell viability. (**B**) Inhibition of HER2 shedding in BT474 cells by CID 3117694. One-way analysis of variance (ANOVA) was used followed by Dunnett post hoc test. The data shown are the mean ± SEM, n = 3 from 3 independent experiments. *****p value < 0.0001, ***p value < 0.001, **p value < 0.01, *p value < 0.05; GI–control inhibitor of ADAM10 and MMPs GI254023X; (**C**) Results of CXCL16 ELISA assay in A549 lung cancer cells. The data shown are the mean ± SEM, n = 3 from 3 independent experiments. One-way analysis of variance (ANOVA) was used followed by Dunnett post hoc test. *****p value < 0.0001, ***p value < 0.001, **p value < 0.01, *p value < 0.05; all compared to DMSO TI. (**D**) Results of syndecan-4 dot blot assay (blot representative of three independent experiments shown).

CID 3117694 did not affect viability of BT474 and HEK293 cells (Fig. 5A), suggesting it can be a useful probe for ADAM10 activity in cell-based systems. CID 3117694 exhibited dose-dependent inhibition of shedding of HER2 from the surface of BT474 cells (Fig. 5B, IC_50_ = 30 ± 2.5 µM), thus validating its potency in a cell-based system against a known ADAM10 substrate. We previously reported that exosite-based inhibition of ADAM17 resulted in the preferential inhibition of a sub-set of ADAM17 substrates^14^. We were interested to see whether CID 31176494 exhibits selectivity towards substrates of ADAM10. CID 3117694 was tested for inhibition of known substrates of ADAM10, CXCL16^26^ and syndecan-4 (unpublished). Syndecan-4 was shown to be cleaved by ADAM17 in response to TNFα/IFN stimulation at 4 hr and 16 hr time points^27^. However, we recently observed that after a longer incubation (e.g., 24 hr) ADAM10 and not ADAM17 is responsible for the cleavage of syndecan-4. This dichotomy of enzymes cleaving syndecan-4 is consistent with differing roles of ADAM10 and 17 in response to various stimuli as reported by others. ADAM17 usually responds rapidly^28^, whereas response of ADAM10 is slower^29^. Based on these considerations, we performed syndecan-4 experiment with 24 hour pre-incubation with TNFα/IFN to test CID 3117694 activity against ADAM10.

Similarly to another ADAM10-selective inhibitor, GI 254023X^11^, CID 3117694 inhibited cytokine-inducible, but not the constitutive shedding of CXCL16 in A549 cells (Fig. 5C). Constitutive shedding of CXCL16 occurs at significantly lower levels than inducible shedding which suggests that inhibition of shedding of CXCL16 by either compound is simply not detectable.

The inhibition potency of CXCL16 shedding by CID 3117694 was similar to that of GI 254023X^11^ despite almost 200-fold difference in *in vitro* potency for ADAM10 (IC_50_ = 5.3 nM and 1.1 µM, for GI 254023X and CID 3117694, respectively). In contrast to CXCL16, syndecan-4 shedding was not inhibited by CID 3117694 (Fig. 5C). Interestingly, GI 254023X inhibited shedding of both CXCL16 and syndecan-4. GI 254023X contains a hydroxamate and inhibits ADAM10 *via* binding of the active site zinc, which results in inhibition of shedding of all ADAM10 substrates. CID 3117694 does not act *via* zinc binding (Fig. 4C,D), which suggests that the lack of inhibition of syndecan-4 shedding could be due to selective exosite-binding rather than the active site binding mode of action of CID 3117694.

### Migration assays

**Figure 6:**
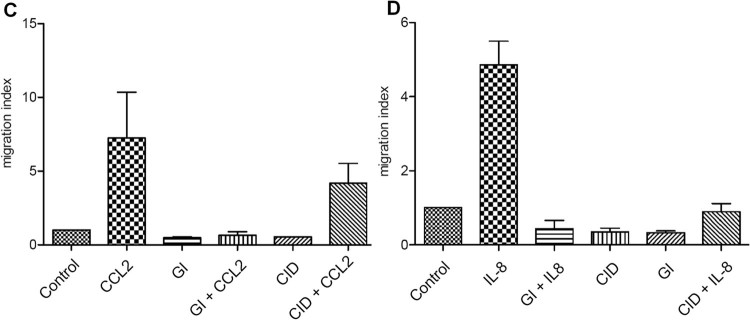
Inhibition of migration by CID 3117694. (**A**) Migration assay with PBMC cells. (**B**) Migration assay with neutrophils. The data shown are the mean ± SEM, n = 3 from 3 independent experiments. One-way analysis of variance (ANOVA) was used followed by Dunnett post hoc test. *****p value < 0.0001, ***p value < 0.001, **p value < 0.01, *p value < 0.05.

ADAM10 was shown to be important for chemokine-induced cell migration^30^. Therefore, we tested CID 3117694 for inhibition of recruitment of inflammatory cells. CID 3117694 was able to inhibit both CCL2-induced migration of peripheral blood mononuclear cells (PBMC) and IL-8-induced migration of primary human neutrophils (Fig. 6A,B). In case of neutrophils, application of CID 3117694 resulted in inhibition of migration to random migration levels, while in the case of PBMC the inhibition of migration was approximately 50%, however, statistical significance was not reached. GI 254023X completely inhibited migration of both PBMC and neutrophils.

### Wound closure assays

**Figure 7:**
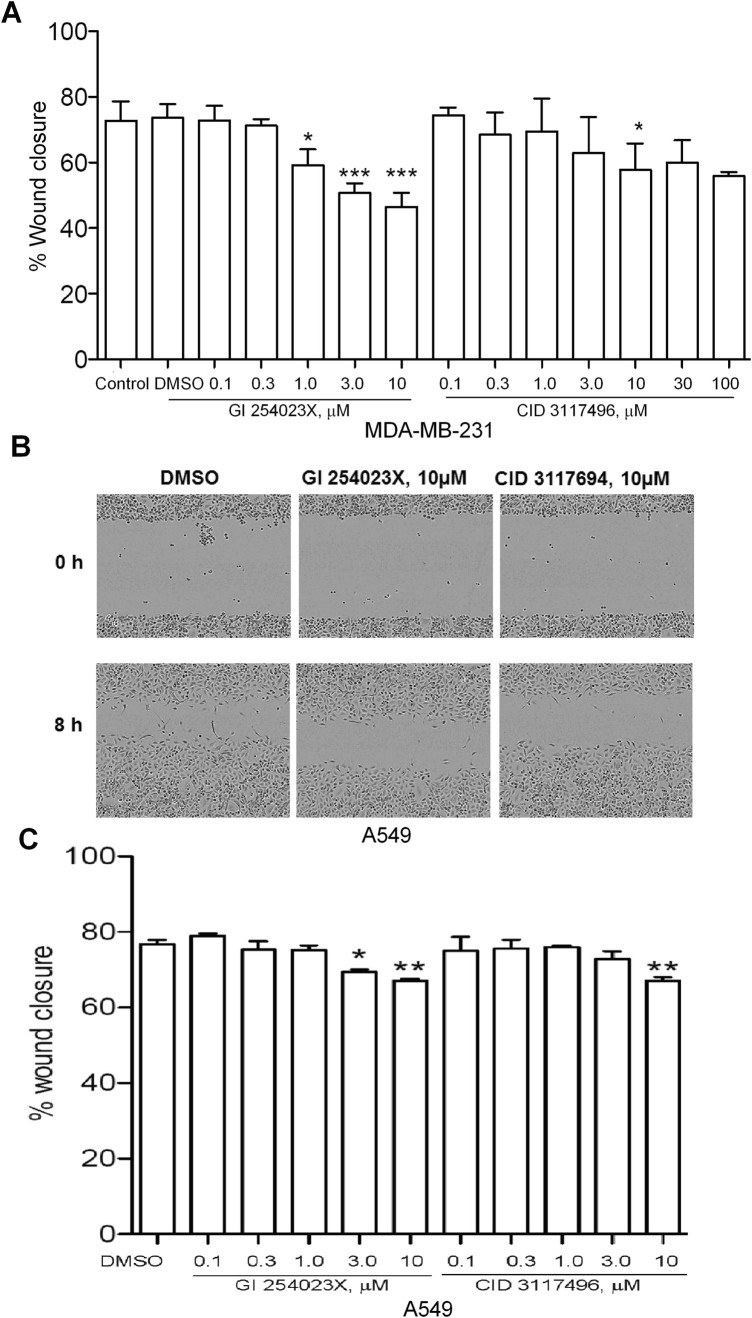
Inhibition of wound closure by CID 3117694. (**A**) Wound closure assay results with MDA-MB-231 cells. (**B**) Representative images of A549 wound closure assay. (**C**) Wound closure assay results with A549 cells. The data shown are the mean ± SEM, n = 3 from 3 independent experiments. One-way analysis of variance (ANOVA) was used followed by Dunnett post hoc test. *****p value < 0.0001, ***p value < 0.001, **p value < 0.01, *p value < 0.05.

Wound healing has been shown to be dependent on ADAM10 and ADAM17 activities^31,32^, but it is not known to what extent either enzyme regulates this process in different tissues. Application of GI 254023X alone cannot differentiate ADAM10 and ADAM17 activity in all cases, and thus we examined whether CID 3117694 is a useful probe of ADAM10 activity in wound healing models. 10 µM CID 3117694 significantly decreased wound healing in MDA-MB-231 and A549 models of wound healing (Fig. 7A–C), suggesting that ADAM10 is involved in this process indicating the usefulness of CID 3117694 in differentiating ADAM10 and ADAM17 involvement in the complex biological processes.

## Discusssion

Exosites have received increased attention for protease, and more specifically metzincin, inhibitor discovery. Cognate substrate-based molecules are the main tools available to the researchers for the screening-based discovery of inhibitors. As has been shown by others^33^ and us^13,23^, exosite-binding substrates can lead to a higher assay sensitivity for exosite inhibitors as compared to active-site substrates only. There is, however, a paucity of substrates that interact with exosites of ADAMs of interest. Knowledge of ADAMs exosites is also limited, as structural information is primarily limited to ADAM catalytic domains^34–36^. Current methods to identify exosite-binding substrates and exosites themselves are laborious^37^ or in early stages of development^38^. We have previously demonstrated that ADAMs can be differentiated based on their ability to hydrolyze glycosylated substrates^13^. Thus, the ability of glycosylated substrates to interact with exosites of ADAMs can be very useful for inhibitor discovery and help overcome the lack of ADAMs structural data.

In the present work we used a TNFα-based, glycosylated, exosite-binding substrate to conduct uHTS to discover non-zinc-binding inhibitors of ADAM10 and ADAM17. While no selective inhibitors of ADAM17 were identified, we discovered a novel non-zinc-binding inhibitor of ADAM10, which is, to our knowledge, a “first-in-class” inhibitor of ADAM10. This compound preferentially inhibited hydrolysis of an exosite-binding glycosylated substrate as compared to an active site-binding-only non-glycosylated substrate, which further corroborates existing evidence of higher selectivity of exosite-binding substrates.

We hypothesize that the lead compound competes with the sugar moiety of glycosylated substrate thereby preventing ADAM10 binding and hydrolysis. Since non-glycosylated substrate does not have the sugar moiety, the lead compound does not compete well with non-glycosylated substrate resulting in low potency of inhibition.

To our knowledge, this is the first report about utilization of a glycosylated protease substrate for an uHTS campaign. Combined with the previously reported discovery of a non-zinc-binding inhibitor of ADAM17 using the same glycosylated substrate^13^, it appears that the glycosylated substrate approach can be used and potentially expanded to other metzincins and different classes of enzymes. For example, studies using glycosylated substrates of metzincins such as Matrix Metalloproteases (MMPs) or pappalysins or other cell-surface enzymes (e.g. BACE-1) will ascertain the applicability of this approach to a broader spectrum of extracellular proteinases. This novel approach could be of great significance for a probe and drug discovery for the wide variety of targets for many therapeutic indications.

Enzymes can have multiple exosites^39,40^ which interact with different substrates. Therefore, targeting different exosites can lead to the discovery of substrate-selective inhibitors (SSIs)^41,42^. We have previously reported that an exosite inhibitor of ADAM17 demonstrated such a substrate selectivity^14^, which suggests the possibility of multiple exosites in the ADAM17 structure. In the present studies we have observed a similar tendency of the exosite-binding inhibitor CID 3117694 for selective inhibition of ADAM10 substrates as evidenced by experiments with both synthetic (glycosylated versus non-glycosylated peptides) and native (syndecan-4 versus CXCL16 and HER2) substrates. The difference in the inhibition of shedding of HER2, syndecan-4 and CXCL16 could potentially be explained in terms of differential glycosylation of these cell surface proteins. CXCL16 is highly modified with mucin-like O-glycosylation containing galactose-N-acetylgalactosamine (Gal-GalNAc) as a part of its core structure within its stalk region where the cleavage by ADAM10 occurs^26,43^. In contrast to CXCL16, syndecan-4 is O-glycosylated by heparan sulfate in three positions^44,45^ and HER2 is N-glycosylated in seven positions^46–48^. The substrate that was used to discover CID 3117694 is O-glycosylated with galactose-N-acetylgalactosamine (Gal-GalNAc) (Fig. 1A), which suggests that CID 3117694 inhibits CXCL16 shedding by preventing its binding to the Gal-GalNAc-binding exosite in ADAM10 structure. The lack of inhibition of syndecan-4 shedding by CID 3117694 is potentially due to the fact that it cannot compete with heparan sulfate moieties which are much larger than Gal-GalNAc. Weak inhibition of HER2 shedding could be explained by the size difference between N-Acetylglucosamine (GlcNAc, monosaccharide) found on HER2 and N-acetylgalactosamine (Gal-GalNAc, disaccharide) found on CXCL16. Another possible explanation is the location of glycosylated residue in relation to the cleavage site. In case of the synthetic glycosylated substrate Gal-GalNAc is only four residues away from the cleavage site (Fig. 1A) which is also likely the case with heavily O-glycosylated CXCL16, whereas in HER2 the most proximal to the cleavage site (^642^PAEQR~ASP^650^)^49^ glycosylation N^629^ is approximately 20 residues away.

Another interesting detail is that despite almost 200-fold difference in *in vitro* potency for ADAM10, GI 254023X and CID 3117694 exhibited similar potency in inhibition of CXCL16 shedding. This corroborates the idea that the type of sugar present on the ADAM10 substrate can affect the potency of the inhibitor that competes with a sugar moiety for the binding to the exosite of ADAM10. These results suggest a possibility of new approaches for inhibitor discovery whereby multiple new substrates carrying different carbohydrates can be designed to bind to different carbohydrate-binding exosites of the same enzyme to drive discovery of SSIs targeting different exosites.

The findings presented herein validate our original hypothesis that targeting exosites of ADAM10 can be used to obtain highly desirable enzyme isoform- and substrate-selective inhibitors. Results of the present study in combination with previously published findings by our group that exosite-binding glycosylated substrate can assist in the discovery of non-zinc-binding inhibitors of ADAM17^13^ strongly suggest that our approach is applicable to probe and drug discovery for ADAMs. Additionally, CID 3117694 can be used as a probe of the biological activity of ADAM10 in various *in vitro* and, potentially*, in vivo* systems.

## Materials and Methods

### Reagents

MMP-1, MMP-2, MMP-8, MMP-9, MMP-10, MMP-13, MMP-14, ADAM10, and ADAM17 were purchased from R&D Systems (cat # 901-MP, 902-MP, 908-MP, 911-MP, 910-MP, 511-MM, 918-MP, 936-AD, and 930-ADB, respectively). All common chemicals were purchased from Sigma. Marimastat was purchased from Tocris (cat# 2631), actinonin was from Sigma-Aldrich (cat# 01809). Hybricare medium was from ATCC (ATCC® 46-X™).

### Synthesis and structural characterization of E(Edans)-PLAQAVRSS^G^S-K(Dabcyl) and E(Edans)-PLAQAVRSSS-K(Dabcyl)

Using Fmoc-protected TF(α1-O)Ser 8 building block^50^, we undertook the synthesis of E(Edans)-PLAQAVRSS^G^S-K(Dabcyl) substrate for ADAM10 and ADAM17 uHTS campaigns. Additionally, we synthesized a non-glycosylated version, E(Edans)-PLAQAVRSSS-K(Dabcyl), to be used as a control in the follow-up studies.

Peptide-resin assembly was performed on a Protein Technology PS3 Peptide Synthesizer by using Tentagel S RAM resin (Advanced ChemTech, Louisville, KY) with an initial load of 0.26 mmol/g. Fmoc-Lys(Dabcyl)-OH and Fmoc-Glu(Edans)-OH were obtained from AnaSpec (Fremont, CA).

Standard Fmoc chemistry was used throughout with a 4-fold molar excess of Fmoc-amino acids, and 2-(6-chloro-1H-benzotriazole-1-yl)-1,1,3,3-tetramethylaminium hexafluorophosphate (HCTU) and 1-hydroxybenzotriazole (HOBt) as coupling reagents. Fmoc-Glu(Edans)-OH was coupled manually in 3-fold molar excess in the presence of benzotriazol-1-yl-oxytripyrrolidinophosphonium hexafluorophosphate (PyBOP) coupling reagent and diisopropylethylamine (DIPEA). The pentafluorophenyl ester of the Fmoc-glycoamino acid was coupled manually in 1.5-fold molar excess to reduce consumption of this amino acid in the presence of DIPEA.

The resulting peptides were cleaved from the resin using thioanisole-water-trifluoroacetic acid (5:5:90) for 2 h. Cleavage solution was extracted with methyl *tert*-butyl ether prior to purification. Deacetylation of the sugar hydroxyl groups was accomplished by a treatment with 0.01 M NaOH for 15 min. Reversed-phase high-performance liquid chromatography (RP-HPLC) purification was performed on a 1260 Infinity Agilent Technologies liquid chromatography system with a Grace Vydac monomeric C18 column (250 × 22 mm, 10 µm, 120 Å) at a flow rate of 20.0 mL/min. Eluents were 0.1% TFA in water (A) and 0.1% TFA in acetonitrile (B). The elution gradient was 0% B for the first 5 min followed by 0–50% B in 60 min. Detection was at λ = 214 nm. Fractions were analyzed by matrix-assisted laser desorption/ionization time-of-flight mass spectrometry (MALDI-TOF MS) and by analytical RP-HPLC.

Analytical RP-HPLC was performed on a 1260 Infinity Agilent Technologies liquid chromatograph equipped with a Grace Vydac monomeric C18 monomeric column (250 × 4.6 mm, 5 µm, 120 Å). Eluents were 0.1% TFA in water (A) and 0.1% TFA in acetonitrile (B). The elution gradient was 0–50% B in 20 min with a flow rate of 1.0 mL/min. Detection was at λ = 214, 360 and 460 nm. MALDI-TOF MS was performed on an Applied Biosystems Voyager MALDI-TOF-DETM STR mass spectrometer using α-cyano-4-hydroxycinnamic acid matrix. The determined peptide mass values were:

$${\rm{Non}}-{\rm{glycosylated}}\,{\rm{substrate}}\,{[{\rm{M}}+{\rm{H}}]}^{+}=1772.42\,{\rm{Da}}\,({\rm{expected}},\,1770.07\,{\rm{Da}})$$

$${\rm{Glycosylated}}\,{\rm{substrate}}\,{[{\rm{M}}+{\rm{Na}}]}^{+}=2160.18\,{\rm{Da}}\,({\rm{expected}},\,2157.40\,{\rm{Da}})$$

As a result of this effort, 300 mg of glycosylated substrate (E(Edans)-PLAQAVRSS^G^S-K(Dabcyl)) was successfully synthesized.

### Meprins expression protocol

Recombinant human meprin α and meprin β were expressed using the Bac-to-Bac expression system (Gibco Life Technologies, Paisley, UK) as described before^51,52^. Media and supplements were obtained from Gibco Life Technologies. Recombinant Baculoviruses were amplified in adherently growing *Spodoptera frugiperda* (*Sf*)9 insect cells at 27 °C in Grace’s insect medium supplemented with 10% fetal bovine serum, 50 units/mL penicillin and 50 μg/ml streptomycin. Protein expression was performed in 500 mL suspension cultures of BTI-TN-5B1-4 insect cells growing in Express Five SFM supplemented with 4 mM glutamine, 50 units/mL penicillin, and 50 μg/mL streptomycin in Fernbach-flasks using a Multitron orbital shaker (INFORS AG, Bottmingen, Switzerland). Cells were infected at a density of 2 × 10^6^ cells/mL with an amplified viral stock at a multiplicity of infection (MOI) of ~10. Protein expression was stopped after 72 h, and recombinant meprins were further purified from the media by ammonium sulfate precipitation (60% saturation) and affinity chromatography (Streptactin for Strep-tagged meprin α and Ni-NTA for His-tagged meprin β). Meprins were activated by trypsin, which was removed afterwards by affinity chromatography using a column containing immobilized chicken ovomucoid, a trypsin inhibitor.

### ADAM10 and ADAM17 uHTS assays

Both assays followed the same general protocol. 2.5 µL of 2x enzyme solution (20 nM) in assay buffer (10 mM Hepes, 0.001% Brij-35, pH 7.5) were added to solid bottom black 1536 plates (Greiner, cat# 789075). Next, test compounds and pharmacological controls were added to corresponding wells using a 1536 pin tool device (V&P Scientific, San Diego). After 30 min incubation at RT, the reactions were started by addition of 2.5 µL of 2x solutions of glycosylated substrate (20 µM). Reactions were incubated at RT for 2 h, after which the fluorescence was measured using Perkin Elmer Viewlux multimode microplate imager (λ_excitation_ = 360 nm, λ_emission_ = 460 nm). Final concentration of test compounds in assays was 7.0 µM. Primary uHTS campaigns for ADAM10 and ADAM17 were performed on 370,276 compounds from the MLPCN collection^20^.

### uHTS campaign

The miniaturized 1536-well plate format ADAM10 and ADAM17 assays were used to screen a collection of ≈370,000 compounds (MLSMR library, provided by the NIH) on the automated Kalypsys/GNF platform at the Scripps Research Institute Molecular Screening Center (SRIMSC, Jupiter, FL. More details at http://hts.florida.scripps.edu/). Detailed protocols and results of this screening effort are publicly available on the PubChem website (https://pubchem.ncbi.nlm.nih.gov) under Assay ID (AID) 720583 and 743013 for ADAM10 and ADAM17, respectively. Both uHTS campaigns were run separately but in a similar manner. Briefly, the first step was the primary screen of all MLSMR’s test compounds as singlicate against the ADAM10 or ADAM17 target at a final concentration of 7.0 μM. Next, compounds selected as primary hits were cherry-picked and retested in triplicate against the primary screen target and its anti-target (ADAM17 for the ADAM10 screening effort, and vice-versa) at the same final concentration of 7.0 μM. The final step was the titration of select hits as 10-point, 1:3 serial dilutions in both the target and anti-target assay, starting at a final nominal concentration of 69.5 μM. For all the aforementioned assays, marimastat, at a final concentration of 1 µM, was used as a positive control and reference for 100% inhibition. Wells treated with DMSO only were used as negative controls and 0% inhibition reference. The percent inhibition of each well was then normalized as follows:

$$ \% \text{\_}{\rm{Inhibition}}=({\rm{RFU}}\text{\_}{\rm{Test}}\text{\_}{\rm{Compound}}-{\rm{MedianRFU}}\text{\_}{\rm{Low}}\text{\_}{\rm{Control}})/\\ \phantom{\rule{7em}{0ex}}({\rm{MedianRFU}}\text{\_}{\rm{High}}\text{\_}{\rm{Control}}-{\rm{MedianRFU}}\text{\_}{\rm{Low}}\text{\_}{\rm{Control}})\ast 100$$

where “Test_Compound” refers to wells containing test compound, “High_Control” is defined as wells treated with 1 µM marimastat (*n* = 24) and “Low_Control” as wells containing DMSO only (*n* = 24). All data generated during this effort were uploaded to the SRIMSC’s institutional screening database (Assay Explorer, Symyx). Sample to background (S/B) ratios, as well as Z and Z′ values were calculated on a per-plate basis as described before^53^. Curve fitting and resulting IC_50_ determinations were performed as previously reported^54^.

### MMP assays

All assays followed the same general protocol. 5 µL of 2x enzyme solution (5 nM) in assay buffer (50 mM Tricine, 50 mM NaCl, 10 mM CaCl_2_, 0.05% Brij-35, pH 7.5) were added to solid bottom black 384 plates (Nunc, cat# 264705). Next, test compounds and pharmacological controls were added to corresponding wells using a 384 pin tool device (V&P Scientific, San Diego). After 30 min incubation at RT, the reactions were started by addition of 5 µL of 2x solutions of MMP substrate (R&D Systems cat#: ES010, 20 µM). Reactions were incubated at RT for 1 h, after which the fluorescence was measured using the Synergy H4 multimode microplate reader (Biotek Instruments) (λ_excitation_ = 324 nm, λ_emission_ = 390 nm).

### Meprin α and β assays

Both assays followed the same general protocol^53^. 5 µL of 2x enzyme solution (2.6 and 0.1 nM for meprin α and β, respectively) in assay buffer (50 mM Hepes, 0.01% Brij-35, pH 7.5) were added to solid bottom black 384 low volume plates (Nunc, cat# 264705). Next, 75 nL of test compounds or pharmacological control (actinonin) were added to corresponding wells using a 384 pin tool device (V&P Scientific, San Diego). After 30 min incubation at RT, the reactions were started by addition of 5 µL of 2x solutions of substrates (20 µM, mepin α Mca-YVADAPK-K(Dnp); and for meprin β Mca-EDEDED-K(Dnp). Reactions were incubated at RT for 1 h, after which the fluorescence was measured using the Synergy H4 multimode microplate reader (Biotek Instruments) (λ_excitation_ = 324 nm, λ_emission_ = 390 nm).

Three parameters were calculated on a per-plate basis: (a) the signal-to-background ratio (S/B); (b) the coefficient for variation [CV; CV = (standard deviation/mean) × 100)] for all compound test wells; and (c) the Z- or Z′-factor^55^. Z takes into account the effect of test compounds on the assay window, while Z′ is based on controls.

### HPLC-based ADAM10 and ADAM17 assays

Both assays followed the same general protocol. 30 µL of 3x enzyme solution (30 nM) in assay buffer (10 mM Hepes, 0.001% Brij-35, pH 7.5) were added to solid bottom black 384 plates. Next, 30 µL of 3x test compounds and pharmacological controls were added to corresponding wells. After 30 min incubation at RT, the reactions were started by addition of 30 µL of 3x solution of glycosylated substrate (30 µM). Reactions were incubated at RT for 2 h and then quenched with 10 µL of 500 µM EDTA. Analytical RP-HPLC was performed on a 1260 Infinity Agilent Technologies liquid chromatograph equipped with an Agilent Poroshell 120 column. Eluents were 0.1% TFA in water (A) and 0.1% TFA in acetonitrile (B). The elution gradient was 0–98% B in 15 min with a flow rate of 1.0 mL/min. Detection was at λ = 214, 360, and 460 nm.

### Determination of inhibition type and mechanism

In order to determine true inhibition modality and affinity of CID 3117694, kinetic assays were performed with the range of substrate (0.5–5 [S]/K_M_) and inhibitor concentrations (1.56–50 µM) in quadruplicate. Reactions were allowed to run for 6 h. Progress curve data were fitted using a non-linear regression model for time-dependent inhibition (Equation 1) using MatLab software.

$$[P]={v}_{s}t+\frac{{v}_{i}-{v}_{s}}{{k}_{obs}}\ast [1-\exp (-{k}_{obs}t)]$$

**Equation 1.** V_i_ – initial velocity, V_s_ – steady state velocity, *k*_obs_ – rate constant for the interconversion between V_i_ and V_s_.

Obtained *k*_obs_ values were plotted versus [I] to determine whether the inhibitor binding conforms to a 1- or 2-step mechanism. Resulting curve of best fit was hyperbolic (R^2^ = 0.997) suggesting the two-step binding model; therefore, the data were fitted to Equation 2 to obtain the apparent potency of CID 3117694.

$${k}_{obs}={k}_{off}\,(\frac{1+\frac{[I]}{app{K}_{i}^{\prime} }}{1+\frac{[I]}{app{K}_{i}}})$$

**Equation 2.**_app_ *K*_*i*_ = apparent potency at initial velocity, *V*_*i*_; _app_ *K*_i_* = apparent potency at steady-state velocity, *V*_*s*_;

To obtain true potency, Equation 3 was solved for *K*_*i*_ and *K*_*i*_ *′*. Resulting ratio of K_i_′/K_i_ termed α (alpha) was used to determine mechanism of inhibition. In order to confirm the true binding modality, *k*_*obs*_ was plotted versus [S]/K_M_.

$$ap{p}^{K}i=\frac{[S]+{K}_{M}}{\frac{{K}_{M}}{{K}_{i}}+\frac{[S]}{{K}_{i}^{\prime} }}$$

**Equation 3.** *K*_*i*_ = binding affinity for free enzyme; ${K}_{i}^{\prime}$= binding affinity for enzyme-substrate complex.

### Dual inhibition studies

5 µL of 4X ADAM10 solution (40 µM) were added to solid bottom black 384 low volume plates (Nunc, cat# 264705). Next, 5 µL 10-point 1:3 dilution of acetohydroxamic acid (AHA) or assay buffer (final assay concentrations 1 µM–83 µM) were added. 5 µL of 4X CID 3117694 were added to the wells containing AHA such that the final assay concentrations of CID 3117694 were 0.275, 0.55, 1.1, 2.2, and 4.4 µM. Enzyme-inhibitor mixtures were incubated for 1 h at RT after which 5 µL of 4X glycosylated substrate was added to all wells. Reactions were incubated at RT for 2 h, after which the fluorescence was measured using the Synergy H4 multimode microplate reader (λ_excitation_ = 360 nm, λ_emission_ = 460 nm). IC_50_ values were calculated by fitting normalized data to sigmoidal log vs. response equation utilizing non-linear regression analysis from GraphPad Prizm 6.

Multi-drug combination dose-effect analysis using Compusyn software (ComboSyn, Inc. 599 Mill Run, Paramus, NJ, 07653, USA) was utilized to determine whether inhibitor combinations produced synergy, antagonism, or no effect as compared to a single inhibitor. Combination Index values were obtained using Combination Index (CI) Equation of Chou-Talalay^56^ in Compusyn software (ComboSyn, Inc. 599 Mill Run, Paramus, NJ, 07653, USA) and a normalized isobologram was constructed:

$$\frac{{(D)}_{1}}{{(Dx)}_{1}}+\frac{{(D)}_{2}}{{(Dx)}_{2}}=CI$$

**Equation 4.** Combination Index Equation for Two Drugs.

In the denominators, (Dx)_1_ is the doses of Drug_1_ alone that inhibits x% while (Dx)_2_ is the dose of Drug_2_ alone that inhibits x%. In the numerators, (D)_1_ is the portion of Drug_1_ in combination (D)_1_ + (D)_2_ also inhibits x%. Effect was determined according to the Table 1 (from ref. 57).

### Cell toxicity studies

Test compounds were solubilized in 100% DMSO and added to polypropylene 384 well plates (Greiner cat# 781280). 1,250 of BT474 or HEK293 cells were plated in 384-well plates in 8 µL of serum-free media (HybriCare for BT474, EMEM for HEK293). Test compounds and pharmacological assay control (lapatinib) were prepared as 10-point, 1:3 serial dilutions starting at 10 mM, then added to the cells using the pin tool mounted on Biomek NX^P^. Plates were incubated for 72 h at 37 °C, 5% CO_2_ and 95% RH. After incubation, 8 µL of CellTiter-Glo® (Promega cat# G7570) was added to each well, and incubated for 15 min at room temperature. Luminescence was recorded using a Biotek Synergy H4 multimode microplate reader. Viability was expressed as a percentage relative to wells containing media only (0%) and wells containing cells treated with DMSO only (100%). Three parameters were calculated on a per-plate basis: (a) the signal-to-background ratio (S/B); (b) the coefficient for variation [CV; CV = (standard deviation/mean) × 100)] for all compound test wells; and (c) the Z′-factor. IC_50_ values were calculated by fitting normalized data to sigmoidal log vs. response equation utilizing non-linear regression analysis from GraphPad Prizm 6.

### HER2 shedding inhibition

To determine the effect of CID 3117694 on the release of HER2, an AlphaLISA soluble HER2 assay kit was utilized (Perkin Elmer # AL234 C/F). Briefly, 20,000 BT474 (HER2 positive) breast cancer cells were seeded in 200 µL of complete Hybricare media in 96 well plates. After 4 h, media was aspirated and CID 3117694 (3.125–100 µM) and controls (50 µM marimastat and GI 254023X)^11^ were added in serum-free media. Cells were incubated for 48 h at 37 °C with 5% CO_2_ and under a humidified atmosphere. After incubation, 5 µL of supernatant was transferred into white opaque 384 well plate for HER2 AlphaLISA assay.

### CXCL16 and syndecan-4 shedding inhibition

A549 cells were seeded in 12-well plates and grown to confluency in DMEM supplemented with 10% FBS and 1% P/S. Subsequently, cells were starved serum-free for 4 h, incubated for 1 h with GI 254023X or CID 3117694 (concentrations as indicated) or DMSO (vehicle, 0.1%) and afterwards stimulated with 10 ng/mL TNF and 10 ng/mL IFN for 24 h or left untreated in the presence of inhibitors or DMSO. Supernatants (500 µL) were harvested and centrifuged for 10 min at 16100 g and 4 °C. Serum levels of CXCL16 were measured using the DuoSet human CXCL16 Elisa Kit (R&D systems, Wiesbaden, Germany). Samples were stored at −80 °C and diluted 2-fold after careful thawing. The standard ranged from 0 to 25,000 pg/mL with a detection limit of 391 pg/mL. For detection of soluble syndecan-4, conditioned media (200 µL) were 2-fold diluted in blotting buffer (0.15 M NaCl buffered to pH 4.5 with 50 mM sodium acetate, and with 0.1% Triton X-100), and applied to cationic polyvinylidene difluoride-based membranes (Hybond-N, Amersham Biosciences, Freiburg, Germany) under vacuum in a dot blot apparatus (Amersham Biosciences). The membranes were blocked for 1 h with PBS-T supplemented with 1% bovine serum albumin and 3% milk powder. Human syndecan-4 was detected by incubating membranes overnight at 4 °C with mouse anti-syndecan-4 mAb (0.6 µg/mL) followed by incubation with POD-coupled goat anti-mouse Ab (27 ng/mL in PBS, Jackson Immunoresearch). Chemiluminescence was recorded and quantified using the luminescent image analyzer LAS3000 substrate after addition of ECL advanced substrate (Amersham Biosciences).

### Migration assays

For PBMC or primary human neutrophil chemotaxis assays, transwells with 8 or 5 µm pores were filled with 100 µL of cell suspension (1 × 10^6^ cells in 100 µL DMEM + 0.2% BSA). Lower wells were filled with stimulus solution (3 nM CCL2 or 10 µg/mL CXCL8 (IL8) in assay buffer (RPMI + 0.2% BSA). After incubation of PBMCs for 2 h or neutrophils for 45 min, migrated cells were quantified in the lower well. For inhibitor studies, cells were pre-incubated in migration medium for 30 min and subsequently subjected to the migration experiment. Migrated neutrophils or PBMCs were quantified by measurement of endogenous glucuronidase activity as described^58^.

### Wound closure assays

For live-cell analysis of scratch-induced wound closure, 2.5 × 10^4^ MDA-MB-231 and A549 cells per well were seeded in collagen G (40 µg/mL) (Biochrom AG, Germany) coated 96-well plates near confluence and allowed to grow overnight in standard medium (DMEM supplemented with 10% FBS and 1% P/S). At confluence, cells were pretreated 2 h with mitomycin (10 µg/mL) (Medac, Germany) to block cell proliferation and washed with standard media. Afterwards, cells were incubated in the presence of inhibitor at the indicated concentrations or DMSO (0.1% vehicle). Subsequently, a defined scratch (wound width between 642–767 µm) was performed in each well using the certified Essen Bioscience automated 96-wound maker^TM^ (Essen Biosciences, Hertfordshire, UK) for 96 well-plates. The medium was removed and 100 µL standard medium were added to the wells containing either inhibitor or DMSO. The closure of the wounded area was monitored using the IncuCyte ZOOM system by taking images of each well every 2 h over a period of 24 h. The reduction of wound width was determined over time using the IncuCyte ZOOM microscope software 2014A. Data were expressed as percentage of wound closure after 8 h.
